# *Actinoalloteichus fjordicus* sp. nov. isolated from marine sponges: phenotypic, chemotaxonomic and genomic characterisation

**DOI:** 10.1007/s10482-017-0920-9

**Published:** 2017-08-02

**Authors:** Imen Nouioui, Christian Rückert, Joost Willemse, Gilles P. van Wezel, Hans-Peter Klenk, Tobias Busche, Jörn Kalinowski, Harald Bredholt, Sergey B. Zotchev

**Affiliations:** 1 School of Biology, Newcastle University, Newcastle upon Tyne, NE1 7RU UK; 2 Microbial Genomics and Biotechnology, Center for Biotechnology (CeBiTec), Bielefeld University, 33594 Bielefeld, Germany; 3 Sinskey Laboratory, Department of Biology, Massachusetts Institute of Technology, Cambridge, MA 02142 USA; 4 Molecular Biotechnology, Sylvius Laboratories, Leiden University, 2333BE Leiden, The Netherlands; 5 Xellia Pharmaceuticals, Silurveien 2, 0380 Oslo, Norway; 6 Department of Pharmacognosy, University of Vienna, Althanstraße 14, 1090 Vienna, Austria

**Keywords:** Marine sponges, Actinobacteria, *Actinoalloteichus*, Chemotaxonomy, Comparative genomics, New species, Secondary metabolite biosynthesis gene clusters

## Abstract

Two actinobacterial strains, ADI 127-17^T^ and GBA 129-24, isolated from marine sponges *Antho dichotoma* and *Geodia barretti*, respectively, collected at the Trondheim fjord in Norway, were the subjects of a polyphasic study. According to their 16S rRNA gene sequences, the new isolates were preliminarily classified as belonging to the genus *Actinoalloteichus*. Both strains formed a distinct branch, closely related to the type strains of *Actinoalloteichus hoggarensis* and *Actinoalloteichus hymeniacidonis*, within the evolutionary radiation of the genus *Actinoalloteichus* in the 16S rRNA gene-based phylogenetic tree. Isolates ADI 127-17^T^ and GBA 129-24 exhibited morphological, chemotaxonomic and genotypic features distinguishable from their close phylogenetic neighbours. Digital DNA: DNA hybridization and ANI values between strains ADI 127-17^T^ and GBA 129-24 were 97.6 and 99.7%, respectively, whereas the corresponding values between both tested strains and type strains of their closely related phylogenetic neighbours, *A. hoggarensis* and *A. hymeniacidonis*, were well below the threshold for delineation of prokaryotic species. Therefore, strains ADI 127-17^T^ (= DSM 46855^T^) and GBA 129-24 (= DSM 46856) are concluded to represent a novel species of the genus *Actinoalloteichus* for which the name of *Actinoalloteichus fjordicus* sp. nov. (type strain ADI 127-17^T^ = DSM 46855^T^ = CECT 9355^T^) is proposed. The complete genome sequences of the new strains were obtained and compared to that of *A. hymeniacidonis* DSM 45092^T^ and *A. hoggarensis* DSM 45943^T^ to unravel unique genome features and biosynthetic potential of the new isolates.

**Electronic supplementary material:**

The online version of this article (doi:10.1007/s10482-017-0920-9) contains supplementary material, which is available to authorized users.

## Introduction

Mycelial actinobacteria are the major sources of a variety of bioactive and potentially pharmaceutically useful compounds, some of which are being developed into anticancer agents (Feling et al. 2003; Prudhomme et al. 2008; Bhatnagar and Kim 2010) and antibiotics (Subramani and Aalbersberg 2012; Manivasagan et al. 2014). Recently, marine actinobacteria have become a focus of intensive research since they have been shown to have a remarkable potential for producing unique secondary metabolites not previously isolated from terrestrial actinobacteria (Zotchev 2012). Many such metabolites have antimicrobial activities, suggesting that they may be used as “chemical weapons” to inhibit the growth of organisms competing for nutritional sources (Davies and Ryan 2012). A role for secondary metabolites as signaling molecules has also been proposed after discovering their effect on gene expression of other bacteria exposed to sub-inhibitory concentrations of the compounds (Yim et al. 2007). Whatever their role is, it must be very important for actinobacteria, since genome sequencing typically reveals the presence of 20–40 gene clusters (most of them “silent”) dedicated to the biosynthesis of secondary metabolites in each species (Horinouchi 2007).

The genus *Actinoalloteichus* (Tamura et al. 2000), belonging to the family *Pseudonocardiaceae* (Embley et al. 1988; Stackebrandt et al. 1997) and suborder *Pseudonocardineae* (Labeda et al. 2011), currently encompasses five species with valid names according to LPSN classification (Euzéby 1997), with *Actinoalloteichus cyanogriseus* as the type species (Tamura et al. 2000). Members of the genus *Actinoalloteichus* form substrate and aerial mycelium with an aggregation of straight spore chains. Their cell walls contain *meso*-diaminopimelic acid and are rich in glutamate, glucosamine and alanine. Major cellular fatty acids are iso-C15:0, anteiso-C15:0, iso-C16:0 and C17:0. The predominant menaquinone is MK-9(H4) with the presence of MK-8(H4) and MK-9(H2) in small amounts. The diagnostic phospholipids are phosphatidylethanolamine and phosphatidylmonomethylethanolamine. The G+C content of their DNA is 72–73 mol% (Tamura et al. 2000).

*Actinoalloteichus* strains have been isolated from different habitats: *A. cyanogriseus*, the type species of the genus from a soil sample collected in the Yunnan province of China (Tamura et al. 2000), the halophilic *Actinoalloteichus hoggarensis* from Saharan soil (Boudjelal et al. 2015), *Actinoalloteichus nanshanensis* from the rhizosphere of a fig tree (Xiang et al. 2011), *Actinoalloteichus spitiensis* from a soil in the cold desert of the Indian Himalayas (Singla et al. 2005) and *Actinoalloteichus hymeniacidonis* from the sponge *Hymeniacidon perleve* collected at the inter-tidal beach of Dalian on the Chinese Yellow Sea (Zhang et al. 2006).

So far, several secondary metabolites have been isolated from *Actinoalloteichus* spp., including the cytotoxic macrolactam BE-14106 from soil-derived *A. cyanogriseus* (Fujita et al. 2016), cytotoxic cyclopentenones from *A. nanshanensis* sp. nov. NEAU 119 (Wang et al. 2013), antifungal neomaclafungins from marine *Actinoalloteichus* sp. NPS702 (Sato et al. 2012), and cytotoxic bipyridine and cyanogramide alkaloids from marine-derived *A. cyanogriseus* WH1-2216-6 (Fu et al. 2011, 2014).

The fact that just a few *Actinoalloteichus* isolates studied so far already yielded several novel compounds suggests a substantial potential of this genus for drug discovery. Here, we describe the isolation, morphological, chemotaxonomic and genome-based characterisation of two new representatives of this relatively rare genus from marine sponges collected in the Trondheim fjord (Norway). The comparison of the complete genome sequences of members of the genus *Actinoalloteichus* might unravel unknown gene clusters dedicated to the biosynthesis of bioactive secondary metabolites.

## Materials and methods

### Sampling of marine sponges

Samples of marine sponges *Geodia barretti* and *Antho dichotoma* were collected at the Tautra ridge (Trondheim fjord, Norway, 63′36″N and 10′31″E) using the MINERVA underwater remote-operated vehicle equipped with a net and a robotic manipulator. The collected sponges did not represent endangered or protected species and the samples were collected by a national Norwegian university (Norwegian University of Science and Technology) for research purposes. Sponge samples of approximately 300 g (*G. barretti*, depth 62. 7 m) and 175 g (*A. dichotoma*, depth 60 m) were retrieved and transferred to 1–l sterile plastic containers with screw caps filled with sterile artificial seawater. Samples were kept at 10 °C during transport (about 3 h) and stored at 4 °C until processing.

### Isolation, maintenance and culture conditions

Sponge pieces of approximately 2 cm^3^ were cut out with a sterile scalpel on a sterilised plastic cutting board, transferred to a mortar containing 18 ml sterile artificial seawater with 20% glycerol, and thoroughly ground. The obtained suspensions were transferred to 50 ml plastic tubes with 5 g sterile glass beads and vortexed at maximum speed for 2 min. Dilutions of the processed sponge samples were plated on different agar media as described in Ian et al. (2014).

The two strains, ADI 127-17^T^ and GBA 129-24, were isolated from marine sponges *A. dichotoma* and *G. barretti*, respectively. Both isolates were obtained on ISP2 medium (International *Streptomyces* Project [ISP] medium 2, Shirling and Gottlieb 1966), supplemented with 50% artificial sea water, after 2 weeks of incubation at 28 °C. Strains ADI 127-17^T^ and GBA 129-24 were maintained in 35% (v/v) glycerol at −20 and −80 °C. For most of the chemotaxonomic analyses, freeze-dried biomass for both isolates was obtained from cultures prepared in marine ISP2 broth medium (ISP2 supplemented with 50% artificial sea water) and incubated for 10 days at 28 °C with shaking at 250 revolutions per minute (rpm) while genomic DNAs were extracted from culture grown in 50 ml of 3% TSB medium (Oxoid, UK) prepared in 50% artificial sea water in 250 ml baffled flasks shacked at 250 rpm for 72 h at 28 °C.

### Chemotaxonomy and morphology

Chemotaxonomic and morphological traits for strains ADI 127-17^T^ and GBA 129-24 were determined using standards methods known to be of value in the taxonomic characterisation of the genus *Actinoalloteichus*. Whole cell sugar composition (Lechevalier and Lechevalier 1970), diaminopimelic acid (Staneck and Roberts 1974), menaquinone (Collins 1985) and polar lipid (Minnikin et al. 1984) profiles were detected using chromatographic methods. Fatty acid extracts (Miller 1982; Kuykendall et al. 1988) were analysed and identified by gas chromatography (Agilent 6890N instrument) using the Standard Microbial Identification (MIDI) system and the ACTIN6 database (Sasser 1990). Cryo-scanning electron microscopy was performed according to Celler et al. (2016) for description of the morphological features of both isolates after growth on ISP2 agar plates (in half-strength artificial sea water) at 28 °C for 7 days.

### Phylogeny

Genomic DNAs were extracted using the Qiagen DNeasy Blood and Tissue Kit. The 16S rRNA gene was amplified by PCR using the universal bacterial 16S rDNA primers F27 and R1492 (Lane 1991). Obtained PCR products were cloned into the Qiagen pDrive PCR cloning vector and sequenced using standard M13 vector primers at MWG Biotech (Germany). DNA sequences of almost complete 16S rRNA genes (ca 1400 nucleotides) were compared with those of the type strains of the genus *Actinoalloteichus* available in EzTaxon server database (http://www.ezbiocloud.net/identify) (Chun et al. 2007). Sequences were aligned using Clustal W algorithm (Thompson et al. 1997) and a phylogenetic tree was constructed using the Molecular Evolutionary Genetics Analysis (MEGA) software version 7 (Kumar et al. 2016). The tree was computed using maximum likelihood method (ML) and the resulting tree topology was tested by bootstrap analysis performed with 5000 replicates. The 16S rRNA gene sequences for strains ADI 127-17^T^ and GBA 129-24 have been deposited in Genbank with the accession numbers MF440323 and MF440324, respectively.

### Genome sequencing, assembly and annotation

For isolation of genomic DNA, both isolates were grown aerobically in 50 ml of 3% TSB medium (Oxoid, UK) prepared in 50% artificial sea water in 250 ml baffled flasks at 28 °C, 250 rpm, for 72 h. Genomic DNA was isolated using the Wizard Genomic DNA Purification Kit (Promega, USA) from approximately 2 g of mycelium (wet weight) using the manufacturer’s protocol with the following modification: the clarified lysate prior to precipitation of DNA with isopropanol was extracted once with ½ volume of a 1:1 mixture of phenol/chloroform (pH 8.0). For sequencing and assembly, an approach that was shown to provide high quality data for actinobacterial genomes (Rückert et al. 2015). For each of the two isolates, two libraries were prepared: a WGS library using the Illumina-Compatible Nextera DNA Sample Prep Kit (Epicentre, USA) and a 5 k MatePair library using the Nextera Mate Pair Sample Preparation Kit, both according to the manufacturer’s protocol. All libraries were sequenced in 2 × 250 bp paired read runs on the MiSeq platform.

For isolate ADI 127-17^T^, a total of 4,487,929 reads were obtained, providing 132.3× coverage of the genome. For strain GBA 129-24, a total of 4,169,978 reads provided 124.7× coverage. Reads were assembled using the Newbler assembler v2.8 (Roche). The initial Newbler assemblies consisted of 54 contigs in two scaffolds, with a total of 76 contigs larger 100 bp for strain ADI 127-17^T^, and 64 contigs in two scaffolds, with a total of 90 contigs larger 100 bp for strain GBA 129-24. The Phred/Phrap/Consed software package (Ewing and Green 1998; Ewing et al. 1998; Gordon et al. 1998; Gordon 2003) was used for sequence assembly and quality assessment in the subsequent finishing process. Gaps between contigs were closed by manual editing in Consed (for repetitive elements).

Gene prediction and annotation were done using the PGAP pipeline (http://www.ncbi.nlm.nih.gov/genomes/static/Pipeline.html). Genes were identified using GeneMark (Borodovsky et al. 2003), GLIMMER (Delcher et al. 1999) and Prodigal (Hyatt et al. 2010). For annotation, BLAST searches against the NCBI Protein Clusters Database (35) were performed and the annotation was enriched by searches against the Conserved Domain Database (Klimke et al. 2009) and subsequent assignment of coding sequences to COGs. Non-coding genes and miscellaneous features were predicted using tRNAscan-SE (Marchler-Bauer et al. 2009), Infernal (Eddy 2002), RNAMMer (Lagesen et al. 2007), Rfam (Griffiths-Jones et al. 2005), TMHMM (Krogh et al. 2001), and SignalP (Bendtsen et al. 2004). Secondary metabolite biosynthesis gene clusters in the genomes were identified using antiSMASH 3.03 (Weber et al. 2015) and manually analysed using BLAST. The genome project was deposited in the Genomes OnLine Database (Liolios et al. 2010). Sequencing, finishing and annotation were performed at the CeBiTec and the complete genome sequences of strain ADI127-17 and GBA 129-24 have been deposited in GenBank under the accession numbers CP016076 and CP016077- CP016078, respectively. The genome sequence of *A. hoggarensis* DSM 45943^T^ has been deposited in GenBank under the accession number CP022521.

### Digital DNA: DNA hybridization

Digital DNA: DNA hybridization (dDDH) analyses were performed between isolates ADI127-17^T^ and GBA 129-24 and their close neighbours *A. hoggarensis* and *A. hymeniacidonis* using formula 2 of the Genome-to-Genome Distance Calculator server available at DSMZ website (http://ggdc.dsmz.de/distcalc2.php).

## Results and discussion

### *Actinoalloteichus* isolates from marine sponges *A. dichotoma* and *G. barretti*

**Fig. 1:**
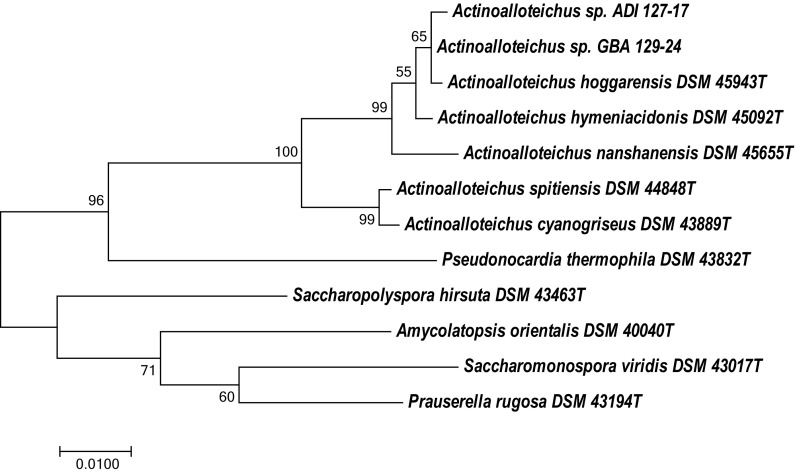
Molecular phylogenetic analysis of *Actinoalloteichus* spp and related actinobacteria using 16S rRNA gene sequences by Maximum Likelihood method. The evolutionary history was inferred by using the Maximum Likelihood method based on the Tamura-Nei model (Tamura and Nei 1993). The tree with the highest log likelihood (−3981.4891) is shown. The percentage of trees in which the associated taxa clustered together is shown next to the branches. Initial tree(s) for the heuristic search were obtained automatically by applying Neighbor-Join and BioNJ algorithms to a matrix of pairwise distances estimated using the Maximum Composite Likelihood (MCL) approach, and then selecting the topology with superior log likelihood value. The tree is drawn to scale, with branch lengths measured in the number of substitutions per site. The analysis involved 12 nucleotide sequences. All positions containing gaps and missing data were eliminated. There were a total of 1358 positions in the final dataset. Evolutionary analyses were conducted in MEGA7 (Kumar et al. 2016)

Two actinobacteria whose 16S rRNA genes displayed >99% identity to the type strains *A. hymeniacidonis* DSM 45092^T^ and *A. hoggarensis* DSM 45943^T^ were isolated from marine sponges *A. dichotoma* (isolate ADI127-17) and *G. barretti* (GBA129-24), respectively, collected at the Trondheim fjord (Norway) as described in “Materials and methods”. Both isolates have been deposited to the DSMZ culture collection with accession numbers DSM 46855^T^ (ADI127-17) and DSM 46856 (GBA129-24), and tentatively assigned to the genus *Actinoalloteichus*. The 16S rRNA gene sequences of DSM 46855^T^ and DSM 46856 were compared to the Ribosomal Database Project database (https://rdp.cme.msu.edu/), confirming the initial taxonomic classification of these isolates. Figure 1 shows the position of *Actinoalloteichus* spp. DSM 46855^T^ and DSM 46856 in a 16S rRNA gene-based phylogenetic tree, which indicates that they are closely related to *A. hoggarensis* DSM 45943^T^. The inferred evolutionary history is supported by the phylogenetic tree generated using the Neighbor-Joining method (Figure S1, Supplementary materials).

While the isolates ADI 127-17 and GBA 129-24 could be cultivated on ISP2 medium supplemented with half-strength artificial sea water, they failed to growth on the same medium prepared with distilled water but with 0, 1, 2, 3, 4 and 5% NaCl. The latter results indicate that but some component(s) in the sea water is/are needed for growth of the two isolates, rather than the osmotic strength.

After 2 weeks of incubation at 28 °C on ISP2 medium prepared with half-strength sea water, strain DSM 46855^T^ displayed black–violet substrate and blue–violet aerial mycelium and produced no diffusible pigment. The phenotype of the strain DSM 45856 grown in the same conditions was very similar, with the only difference being aerial mycelium, which was grey–violet.

The micromorphology of *Actinoalloteichus* sp. strain DSM 46856 examined using a conventional microscope was significantly different from that of strain DSM 46855^T^, as the majority of the spore chains of strain DSM 46856 were embedded within the aerial mycelium (as seen at lower magnification). The aerial hyphae appeared to stick together, indicative of the existence of an extracellular matrix, and knots were regularly observed in the aerial hyphae. The spore chains that were formed were also more homogeneous in spore length. The substrate mycelia of both strains were found to be non-fragmented.

**Fig. 2:**
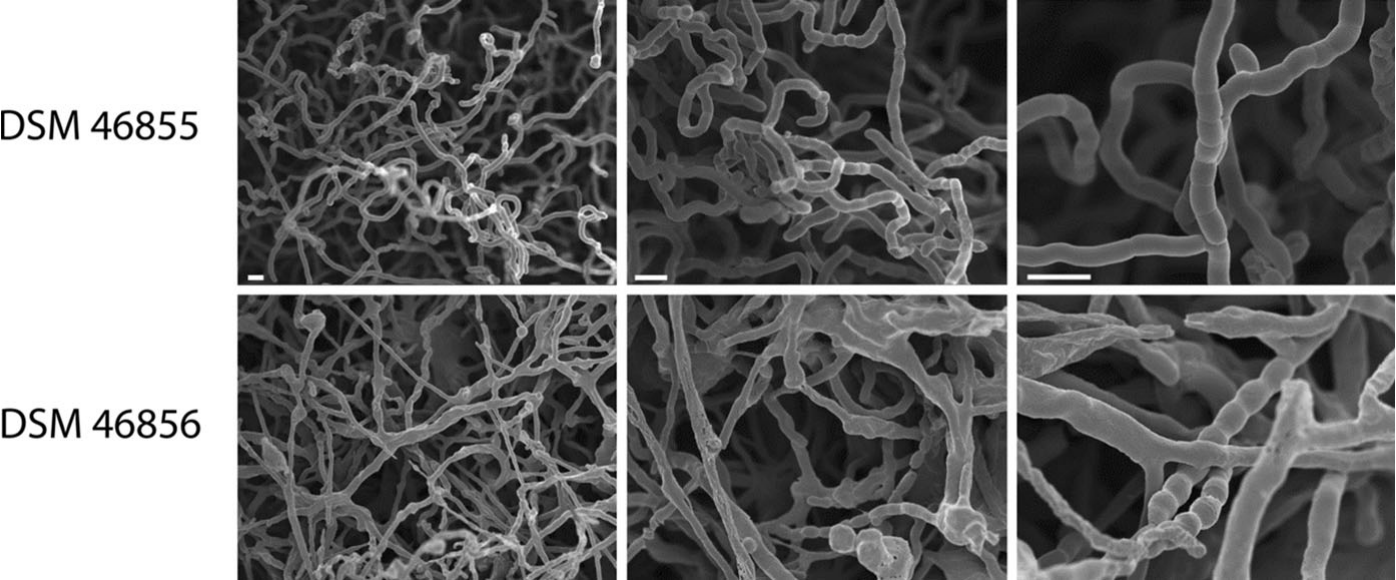
Cryo-scanning electron micrograph of the aerial mycelium of *Actinoalloteichus* spp. DSM 46855^T^ and DSM 46856. The strains were grown on 7 days on ISP2 medium (in half-strength artificial sea water) and then imaged. For each strain, aerial hyphae and spores are shown at three magnifications. *Bar* 2 µm

To examine the micromorphology of the *Actinoalloteichus* isolates in more detail, cultures were grown for 7 days on ISP2 medium (in half-strength artificial sea water) and sporulating aerial hyphae were examined using cryo-scanning electron microscopy (Fig. 2). *Actinoalloteichus* sp. DSM 46855^T^ showed typical sporulation similar to what is observed for streptomycetes, revealing a multitude of aerial hyphae with chains of spores. However, some aerial hyphae branched and the spore sizes were relatively irregular in size, with larger and small spores in a single spore chain. Higher magnification revealed occasional aerial hyphae forming a single septum, which, up to that stage, had failed to progress to sporulation.

### Chemotaxonomy of the new *Actinoalloteichus* isolates

Both new *Actinoalloteichus* isolates were subjected to chemotaxonomic analyses as described in Methods section in comparison with two type species, *A. hymeniacidonis* and *A. hoggarensis*, and the results are presented in Table 1. Analysis of phospholipid content revealed the presence of phosphatidylinositol, phosphatidylglycerol, diphosphatidylglycerol, two glycophospholipids, two unidentified lipids and a glycolipid (Figs. S2–S4, Supplementary materials). None of the following polar lipids were detected: phosphatidylethanolamine, phosphatidylmethylethanolamine or phosphatidylcholine.

**Table 1:** Chemotaxonomic characteristics of the strains DSM 46855^T^, DSM 46856 and the type strains of the closely related species *A. hymeniacidonis* DSM 45092^T^ and *A. hoggarensis* DSM 45943^T^

	DSM 45092^T^	DSM 45943^T^	DSM 46856	DSM 46855^T^
iso-14:0	4	3.7	2.0	2.1
iso6-15:0	6	−	−	−
iso-15:0	−	21.6	8.7	10.6
anteiso-15:0	20	11.7	25.6	20.1
15:0	6	4.1	4.7	5.6
iso-16:1	−	−	−	−
16:1	−	−	−	−
isoG-16:1	6	5.8	2.5	2.2
iso1-16:0	16	−	−	−
iso-16:0	−	18.2	10.9	10.9
16:0	−	−	1.8	1.6
Iso-17:0	−	4.0	1.7	2.0
anteiso-17:0	4	4.2	10.8	9.6
W8C-17:1 = cis9-17:1	19	−	9.1	10.3
17:0	11	8.8	13.6	18.3
MK-8(H4)	−	−	−	3.6
MK-9(H2)	−	−	−	0.4
MK-9(H4)	64	+(54%)^a^	99.7	58.1
MK-9(H6)	23	+(3.2%)^a^	−	4.1
MK-9(H8)	12	−	−	−
MK-10(H4)	−	+(34.2%)^a^	0.4	9.4
Phosphatidylethanolamine	+	+	−	−
Phosphatidylglycerol	+	+	+	+
Phosphatidylinositol	+	+	+	+
Phosphatidylinositol mannoside	+	+	−	−
Glycophospholipids	−	−	+	+
Diphosphatidylglycerol	−	+	+	+
Phosphatidylmonomethyl-ethanolamine	−	−	−	−
Phosphatidyl choline	−	−	−	−
*meso*-A2 pm	+	+	+	+
Galactose, glucose, mannose, ribose, rhamnose (trace) unidentified sugar	+	+	+	+

^a^Absolute values for *A. hoggarensis* DSM 45943^T^ not reported

The same pattern of cell sugars was visualised for both *Actinoalloteichus* spp. DSM 46855^T^ and DSM 46856: galactose, glucose, mannose, ribose, rhamnose (trace) and an unidentified sugar. The major fatty acids were found to be anteiso-15:0 (20.1, 25.6%), 17: 0 (13.6, 18.3%), iso-16:0 (10.9, 10.9%), *cis*9-17:1 (9.1, 10.3%), iso-15:0 (8.7, 10.6%) for strains DSM 46856 and DSM 46855^T^, respectively. DL-diaminopimelic acid was revealed for both isolates. The major abundant quinone was identified as MK-9(H4) for both strains.

According to the chemotaxonomic analyses, the new isolates clearly differed from *A. hymeniacidonis*, being more similar to *A. hoggarensis*. However, major fatty acid composition, menaquinone and phospholipid profiles of *Actin oalloteichus* spp. DSM 46855^T^ and DSM 46856 were significantly different from those of *A. hoggarensis*. For example, unlike *A. hoggarensis*, the new isolates did not have phosphatidylethanolamine in their phospholipid profiles. Also, their fatty acid composition varied from that of the *A. hoggarensis*: they contained considerably less iso-C_14:0_, iso-C_15:0_, isoG-C_16:1_, iso-C_16:0_, and iso-C_17:0_ fatty acids while having significantly more anteiso-C_15:0_, C_16:0_, anteiso-C_17:0_, and C_17:0_ fatty acids.

### Genomics of *Actinoalloteichus* spp. DSM 46855^T^ and DSM 46856

Due to the increasing interest in identifying new actinobacterial species that can be potential sources of novel bioactive secondary metabolites, the new *Actinoalloteichus* isolates were subjected to genome analyses. Sequencing, finishing and annotation were performed, and the complete genome sequences deposited in GenBank with accession numbers CP016076 (DSM 46855^T^), CP016077 (DSM 46856 chromosome) and CP016078 (DSM 46856 plasmid). A summary of the project information is shown in Table 2.

**Table 2:** Genome statistics for *Actinoalloteichus* spp. DSM 46855^T^ and DSM 46856

Attribute	DSM 46855^T^		DSM 46856^T^	
	Value	% of total^a^	Value	% of total^a^
Genome size (bp)	7,120,854	100.00	7,275,385	100.00
DNA coding region (bp)	5,909,200	82.98	6,059,562	83.29
DNA G+C content (bp)	4,990,823	70.09	5,100,026	70.10
DNA scaffolds	1		2	
Total genes	6047	100.00	6264	100.00
Protein-coding genes	5952	98.43	6178	98.63
RNA genes	78	1.29	80	1.28
Pseudo genes	14	0.23	6	0.10
Genes in internal clusters	1025	16.95	1231	19.65
Genes with function prediction	4360	72.10	4412	70.43
Genes assigned to COGs	3500	57.88	3538	56.48
Genes with Pfam domains	4574	75.64	4633	73.96
Genes with signal peptides	297	4.91	298	4.75
Genes with transmembrane helices	1309	21.65	1331	21.25
CRISPR repeats	19		14	

^a^The total is based on either the size of the genome in base pairs or the total number of total genes in the annotated genome

**Fig. 3:**
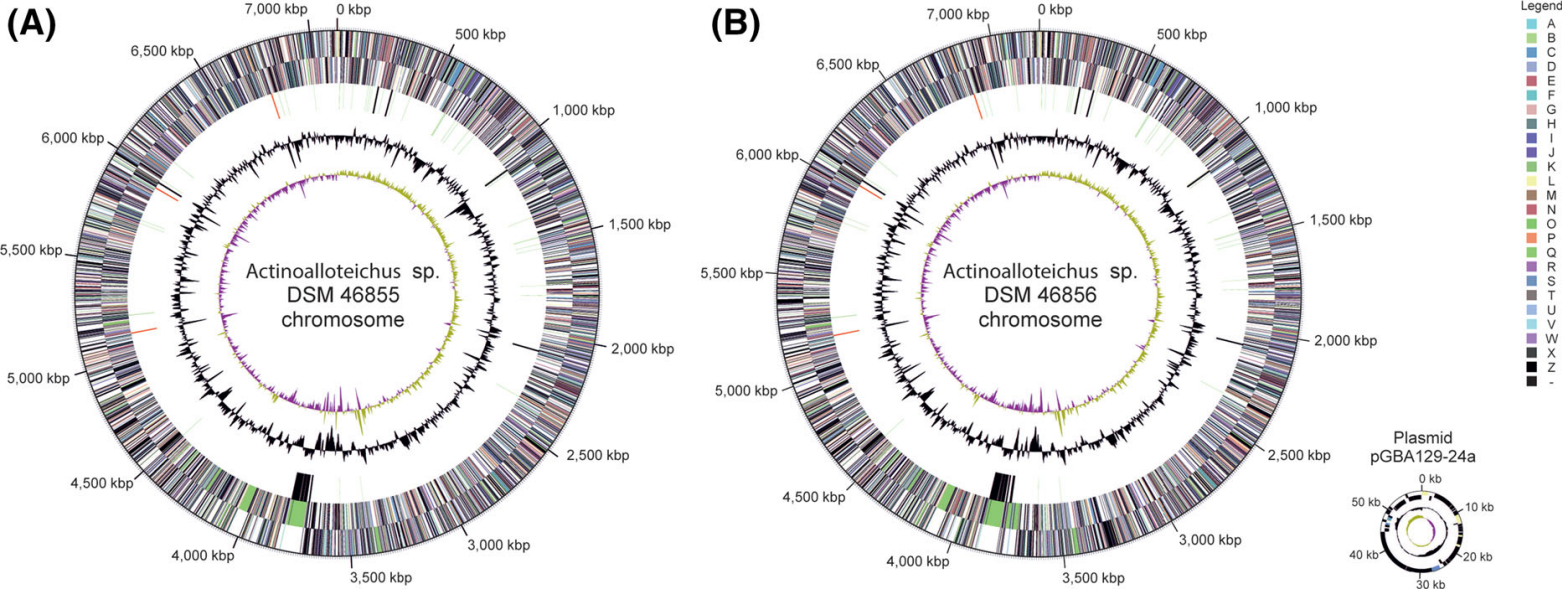
Graphical maps of the chromosomes of **a** *A. fjordicus* DSM 46855^T^ and **b** *A. fjordicus* DSM 46856. From the *outside* to the *center*: Genes on forward strand (*colour* by COG categories), genes on reverse strand (*color* by COG categories—see Table 3), RNA genes (tRNAs *green*, rRNAs *red*, other RNAs *black*), G+C content, G+C skew

The genome of strain DSM 46855^T^ consists of one circular chromosome of 7,120,854 bp (70.09% G+C content), while the genome of strain DSM 46856 consists of one circular chromosome of 7,215,977 bp (70.11% G+C content) and a plasmid of 59,408 bp (69.21% G+C content). Among a total of 6064 predicted genes in the DSM 46855^T^ genome, 5986 appear to be protein coding, while these numbers for DSM 46856 are 6271 and 5986, respectively. In total, 4360 protein-coding genes of DSM 46855^T^ (71.90%) and 4412 protein-coding genes of strain DSM 46856 (70.36%) were assigned a putative function, the remaining were annotated as hypothetical proteins. The properties and the statistics of the genomes are briefly summarised in Tables 2 and 3, and the circular plots of the chromosomes and a plasmid pGBA129-24a detected in strain DMS 46856 are shown in Fig. 3.

**Table 3:** Number of genes in the genomes of *Actinoalloteichus* spp. DSM 46855^T^ and DSM 46856 associated with the general COG functional categories

Code	DSM 46855		DSM 46856		Description
	Value	% age	Value	% age	
J	205	3.44	202	3.27	Translation, ribosomal structure and biogenesis
A	1	0.02	1	0.02	RNA processing and modification
K	466	7.83	471	7.62	Transcription
L	120	2.02	130	2.10	Replication, recombination and repair
B	1	0.02	1	0.02	Chromatin structure and dynamics
D	34	0.57	35	0.57	Cell cycle control, cell division, chromosome partitioning
V	174	2.92	172	2.78	Defense mechanisms
T	196	3.29	198	3.20	Signal transduction mechanisms
M	174	2.92	176	2.85	Cell wall/membrane biogenesis
N	5	0.08	7	0.11	Cell motility
U	30	0.50	31	0.50	Intracellular trafficking and secretion, and vesicular transport
O	141	2.37	140	2.27	Posttranslational modification, protein turnover, chaperones
Z	0	0.00	0	0.00	Cytoskeleton
W	5	0.08	7	0.11	Extracellular structures
C	211	3.55	214	3.46	Energy production and conversion
G	385	6.47	383	6.20	Carbohydrate transport and metabolism
E	334	5.61	335	5.42	Amino acid transport and metabolism
F	98	1.65	98	1.59	Nucleotide transport and metabolism
H	268	4.50	266	4.31	Coenzyme transport and metabolism
I	194	3.26	193	3.12	Lipid transport and metabolism
P	209	3.51	211	3.42	Inorganic ion transport and metabolism
Q	189	3.18	207	3.35	Secondary metabolites biosynthesis, transport and catabolism
R	485	8.15	484	7.83	General function prediction only
S	156	2.62	158	2.56	Function unknown
X	5	0.08	12	0.19	Mobilome: prophages, transposons
–	2564	43.08	2733	44.24	Not in COGs

In order to resolve the ambiguity regarding relatedness of new isolates and *A. hoggarensis* DSM 45943^T^ stemming from phylogenetic analysis of their 16S rRNA genes (Fig. 1), the genome of the latter bacterium was sequenced (Genbank accession number CP022521). Like the genome of *A. hymeniacidonis* DSM 45092^T^ (Schaffert et al. 2016) with 6306 kbp (68.08% G+C content), the genome of *A. hoggarensis* DSM 45943^T^ is significantly smaller than those of the two *Actinoalloteichus* strains DSM 46855^T^ and DSM 46856, consisting of a single, circular chromosome of 6607 kbp with a G+C content of 71.15%.

The significantly different genome sizes and G+C contents provided further evidence that the *Actinoalloteichus* strains DSM 46855^T^ and DSM 46856 represent a distinct novel species. Based on the available genome sequences for *Actinoalloteichus* spp. DSM 46855^T^, DSM 46856, DSM 45092^T^ and DSM 45943^T^, which appear most related based on the 16S rRNA gene sequences, we performed genome-to-genome distance calculations (Table 4). The 16S rRNA gene sequences of *Actinoalloteichus* spp. DSM 46855^T^, DSM 46856 were 99.9% identical to that of the most closely related species, *A. hoggarensis* DSM 45943^T^. However, both DDH and ANI estimates obtained clearly suggest that strains DSM 46855^T^ and DSM 46856 belong to the same species (DHH = 97.6%, ANI = 99.68%), which is distinct from *A. hoggarensis* (DHH = 30.9%, ANI = 85.96% compared with strain DSM 46855^T^). Based on all the analyses described above, we propose that the isolates DSM 48655^T^ and DSM 48656 represent a new species of the genus *Actinoalloteichus*, with the proposed name *Actinoalloteichus fjordicus*. It is notable that *A. fjordicus* is the first representative of the genus *Actinoalloteichus* which appears to be an obligate marine species. The Digital Protologue database (Rosselló-Móra et al. 2017) Taxon Number for strain DSM 46855^T^ is TA00145.

**Table 4:** Data from the genome analyses of four *Actinoalloteichus* spp. using genome-to-genome distance calculator (DSMZ: http://ggdc.dsmz.de/)

Strain	DDH			
	*A. fjordicus* DSM 46855^T^	*A fjordicus* DSM 46856	*A. hoggarensis* DSM 45943^T^	*A. hymeniacidonis* DSM 45092^T^
*A. fjordicus* DSM 46855^T^	100.0%	97.6%	30.9%	24.5%
*A. fjordicus* DSM 46856		100.0%	30.9%	24.5%
*A. hoggarensis* DSM 45943^T^			100.0%	23.9%
*A. hymeniacidonis* DSM 45092^T^				100.0%

Strain	ANI			
	*A. fjordicus* DSM 46855	*A fjordicus* DSM 46856	*A. hoggarensis* DSM 45943T	*A. hymeniacidonis* DSM 45092T
*A. fjordicus* DSM 46855	100.00%	99.68%	85.96%	80.05%
*A fjordicus* DSM 46856		100.00%	85.98%	79.98%
*A. hoggarensis* DSM 45943T			100.00%	79.51%
*A. hymeniacidonis* DSM 45092T				100.00%

## Description of *A. fjordicus* sp. nov.

*Actinoalloteichus fjordicus* sp. nov (fjor’di.cus. N.L. masc. adj. *fjordicus*, referring to a Norwegian fjord, the site of isolation).

Aerobic, Gram-stain positive, catalase positive actinobacterium that forms an extensively branched substrate mycelium that bears aerial hyphae that differentiate into straight chains of smooth surfaced spores of unusual bluish on ISP2 medium. Grows from 20 to 35 °C, optimally at ~28 °C, optimally at ~pH 7.0, and requires at least 50% sea water in the media for growth. Additional cultural and phenotypic features are cited in the text and in Table 1. Chemotaxonomic characteristics are typical of the genus *Actinoalloteichus.*

The type strain, ADI127-7^T^ (= DSM 46855^T^ = CECT 9355^T^), was isolated from a sponge sample of *Antho dichotoma* collected at a depth of 60 m below sea level at the Tautra ridge in the Trondheim fjord in Norway (63′36″N and 10′31″E). The GenBank accession numbers for the 16S rRNA gene sequences of DSM 46855^T^ and the related strain DSM 46856 are MF440323 and MF440324, respectively.

### Gene clusters for biosynthesis of secondary metabolites in the genomes of *A. fjordicus*

All sequenced actinobacterial genomes contain gene clusters for biosynthesis of secondary metabolites, but the information regarding the types of compounds that representatives of the genus *Actinoalloteichus* can produce is scarce. Moreover, to our knowledge, only one biosynthetic gene cluster from *Actinoalloteichus* has been characterised so far (Zhu et al. 2012). Recently, we sequenced the genome of sponge-derived *A. hymeniacedonis*, and described analysis of its secondary metabolite biosynthesis gene clusters, SMBGCs (Schaffert et al. 2016). Considering the above, it was of interest to compare the biosynthetic potential of the latter, as well as terrestrial *Actinoalloteichus* spp. genomes (albeit draft quality) with those of the new isolates.

The genomes of DSM 46855^T^, DSM 46856, *A. hymeniacedonis*, *A. hoggarensis*, *A. cyanogriseus* and *A. spitinensis* were analysed for the presence of SMBGCs using the online version of software antiSMASH 4.0 (Weber et al. 2015). The results of the analysis were manually curated to confirm or edit borders of the clusters, identify closest homologues in the databases based on BLAST searches, and to gain more detailed insight into the biosynthesis of the corresponding compounds. The results of the analysis are presented in Table 5.

**Table 5:** Secondary metabolite biosynthesis gene clusters identified in the genomes of *Actinoalloteichus* sp. DSM 46855^T^, *A. hymeniacidonis* DSM 45092^T^, *A. hoggarensis* DSM 45943^T^, *A. cyanogriseus* DSM 43889 and *A. spitinensis* DSM 44848 using antiSMASH 4.0 software followed by manual curation

No	Gene cluster type	Putative product	Presence in			
			*A*. *hymeniacidonis*	*A. hoggarensis*	*A. spitiensis*	*A. cyanogriseus*
1	Ectoine	**Ectoine**	**+**	**+**	**+**	**+**
*2*	*Nrps-t1pks*	*PK-NRP hybrid, halogenated*	***−***	***−***	***−***	***−***
3	Laderrane	Ladderane	**+**	**−**	**+**	**–**
4	Nrps-t1pks	PK-NRP hybrid, glycosylated	**+**	**+**	**−**	**−**
5	Ectoine	**Ectoine**	**+**	**+**	**+**	**−**
6	Lasso peptide	Lasso peptide	**+**	**+**	**−**	**−**
*7*	*Lantipeptide*	*Lantibiotics, class II*	***−***	***−***	***−***	***−***
8	Terpene	Carotenoid	**+**	**+**	**−**	**−**
9	T2pks	Xanthone	**+**	**+**	**−**	**−**
10	Siderophore	Siderophore	**+**	**+**	**+**	**+**
11	Terpene	Lycopene	**+**	**+**	**−**	**−**
12	Terpene	Carotenoid	**−**	**+**	**−**	**−**
13	Nrps	**Nocardicin**	**−**	**+**	**+**	**+**
*14*	*Nucleoside*	*Peptide-nucleoside*	***−***	***−***	***−***	***−***
15	T1pks	Polyene macrolide, glycosylated	**−**	**+**	**−**	**+**
16	Nrps	Modified dipeptide	**−**	**−**	**+**	**−**
*17*	*Nrps*	*Dapdiamide*	***−***	***−***	***−***	***−***
18	T1pks	20-membered macrolide, glycosylated	**−**	**+**	**−**	**−**
19	T1pks-nrps	**Maduropeptin**	**−**	**−**	**−**	**+**
20	Nrps-t1pks	**Dihydromaltophilin**	**+**	**+**	**+**	**+**
21	T3pks	Unknown	**−**	**+**	**−**	**−**
22	T1pks	Enediyne	**−**	**+**	**−**	**+**
23	Terpene	Unknown	**+**	**+**	**+**	**−**
*24*	*Bacteriocin*	*Bacteriocin*	***−***	***−***	***−***	***−***
*25*	*Amglyccycl*	*Aminocyclitol (salbostatin-like)*	***−***	***−***	***−***	***−***
26	T1pks-t4pks	Unknown	**+**	**+**	**+**	**−**
27	Butyrolactone	γ-butyrolactone	**+**	**−**	**−**	**−**
*28*	*Indole*	*Indole*	***−***	***−***	***−***	***−***

Clusters unique for *Actinoalloteichus* sp. DSM 46855^T^ are italic. Presumed known metabolites are shown in bold font

The new *A. fjordicus* strains were found to harbour 27 identical SMBGCs, the genome of DSM 46855^T^ containing one additional cluster compared to that of DSM 46856, presumptively governing biosynthesis of a peptide-nucleoside compound. Five gene clusters that are likely to be responsible for the biosynthesis of known secondary metabolites, ectoine (two gene clusters, Sadeghi et al. 2014), nocardicin (Gunsior et al. 2004), maduropeptin (Van Lanen et al. 2007) and dihydromaltophilin (Yu et al. 2007) were identified in both DSM 46855^T^ and DSM 46856 genomes. One ectoine biosynthesis gene cluster was found in all the analysed genomes, but the second one was only identified in *A. hoggarensis* and *A. hymeniacedonis*. While the nocardicin gene cluster was present in the genomes of *A. hoggarensis*, *A. cyanogriseus* and *A. spitiensis,* it could not be identified in the *A. hymeniacedonis* genome. The maduropeptin gene cluster was identified in *A. cyanogriseus*, but not in the genomes of other *Actinoalloteichus* species investigated in this study. The dihydromaltophilin gene cluster was identified in all analysed genomes. Compared to other analysed *Actinoalloteichus* spp., seven SMBGCs appeared to be unique for the *A. fjordicus* strains (Table 5). Of those, 4 (clusters 2, 7, 14 and 24) could not be matched to any cluster available in the public databases as of March 2017, suggesting that DSM 46855^T^ and DSM 46856 may be interesting sources of previously unknown natural products. The knowledge on the respective SMBGCs and their putative products will help to identify corresponding compounds, and may assist in their testing and development as potential drug candidates.

## Electronic supplementary material

Below is the link to the electronic supplementary material.

Supplementary material 1 (DOCX 1548 kb)
