# Supplementary material for: Actinoalloteichus fjordicus sp. nov. isolated from marine sponges: phenotypic, chemotaxonomic and genomic characterisation
Source: Antonie Van Leeuwenhoek. 2017 Aug 2;110(12):1705–17. doi: 10.1007/s10482-017-0920-9 (PMC5676828; doi:10.1007/s10482-017-0920-9)
Supplement: Supplementary file 1 — Supplementary material 1 (DOCX 1548 kb) [file 10482_2017_920_MOESM1_ESM.docx]

**Supplementary materials**

***Actinoalloteichus fjordicus* sp. nov. isolated from marine sponges: phenotypic, chemotaxonomic and genomic characterisation**

Imen Nouioui, Christian Rückert, Joost Willemse, Gilles P. van Wezel, Hans-Peter Klenk, Tobias Busche, Jörn Kalinowski, Harald Bredholt, Sergey B. Zotchev

**Figure S1** Molecular Phylogenetic analysis of *Actinoalloteichus* spp. and related actinobacteria using 16S rRNA gene sequences by Neighbor-Joining method. The optimal tree with the sum of branch length = 0.21419219 is shown. The percentage of replicate trees in which the associated taxa clustered together in the bootstrap test (500 replicates) are shown next to the branches. The tree is drawn to scale, with branch lengths in the same units as those of the evolutionary distances used to infer the phylogenetic tree. The evolutionary distances were computed using the Maximum Composite Likelihood method and are in the units of the number of base substitutions per site. The analysis involved 13 nucleotide sequences. All positions containing gaps and missing data were eliminated. There were a total of 1360 positions in the final dataset. Evolutionary analyses were conducted in MEGA7.

**Figure S2** Two-dimensional TLC plates of polar lipids extracted from *Actinoalloteichus fjordicus* ADI 127-17^T^ (A) and *M. fjordicus* GBA 129-24 (B) stained with molybdatophosphoric acid (Sigma P1518) to identify total lipid content. Key: DPG, diphosphatidylglycerol; PG, phosphatidylglycerol; PI, phosphatidylinositol; GPL, glycophospholipid; GL, glycolipid and L_1-2_, lipid. Solvent 1: chloroform: methanol: distilled water (65:25:4); Solvent 2: chloroform : glacial acetic acid: methanol: distilled water (80:12:15:4).

**B**

**A**


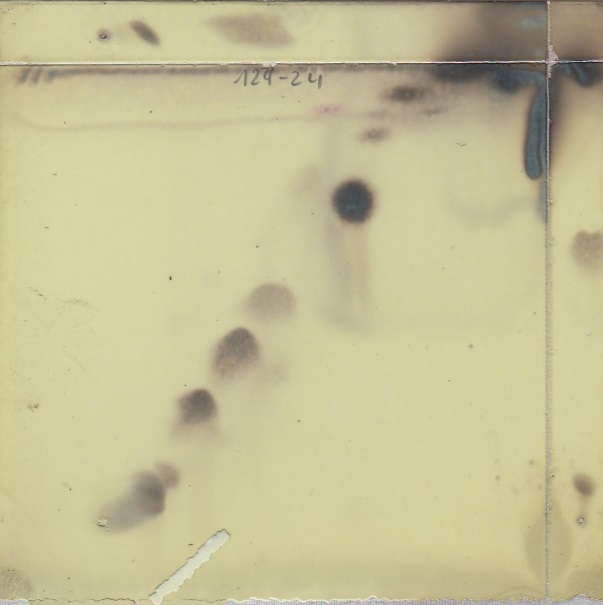


PI

GPL1

GL

PG

DPG

GBA 129-24

GPL2

L1

L2


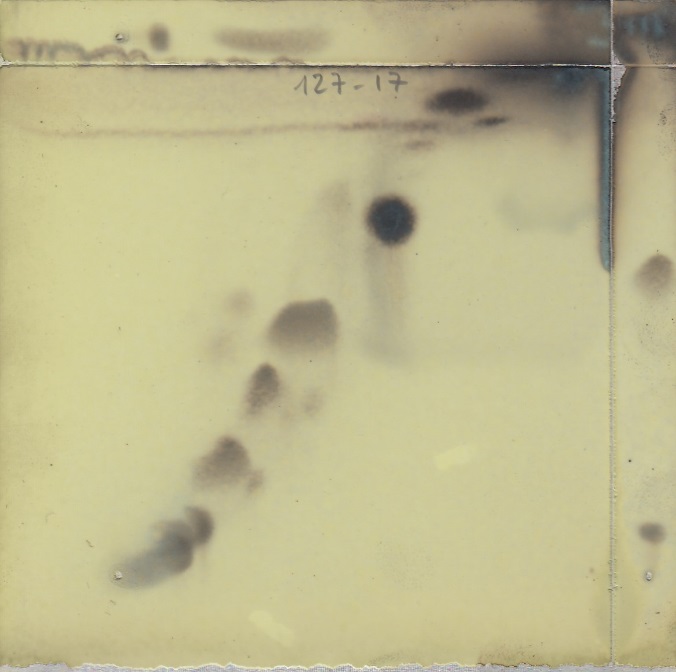


DPG

PG

PI

GPL1

GL

ADI 127-17^T^

GPL2

L1

L2

**Figure S3** Two-dimensional TLC plates of polar lipids extracted from *Actinoalloteichus fjordicus* ADI 127-17^T^ stained with (A) molybdatophosphoric acid (Sigma P1518) to identify total lipid content, (B) 0.2 % Ninhydrin reagent to detect amino-groups, (C) Molybdenum blue (Sigma 119KG123) to identify phospho-groups, (D) α-Naphtho-sulphuric acid to detect glyco-groups, (E) Dragendorff reagent to identify choline-groups. Key: DPG, diphosphatidylglycerol; PG, phosphatidylglycerol; PI, phosphatidylinositol; GPL, glycophospholipid; GL, glycolipid and L_1-2_, lipid. Solvent 1: chloroform: methanol: distilled water (65:25:4); Solvent 2: chloroform : glacial acetic acid: methanol: distilled water (80:12:15:4).


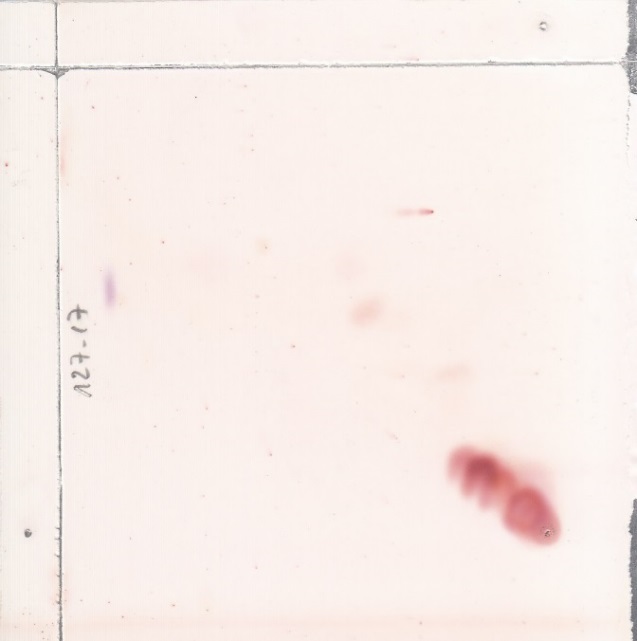


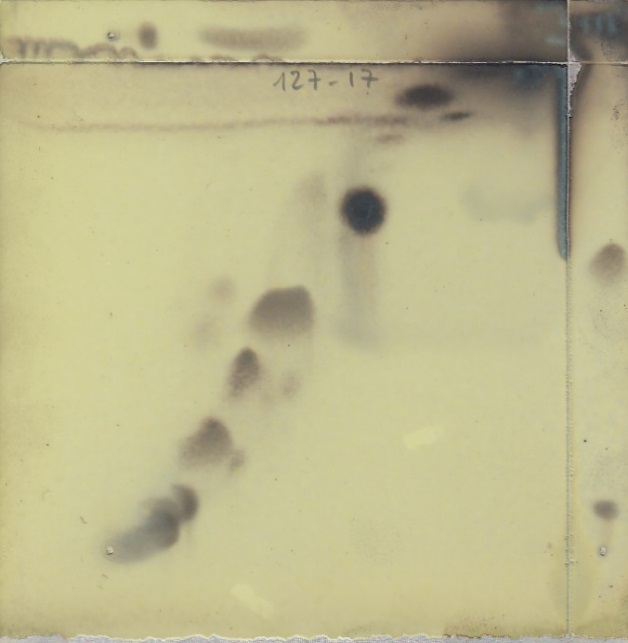


DPG

PG

PI

GPL1

GL

GPL2

L1

L2

ADI 127-17^T^

ADI 127-17^T^

A

B


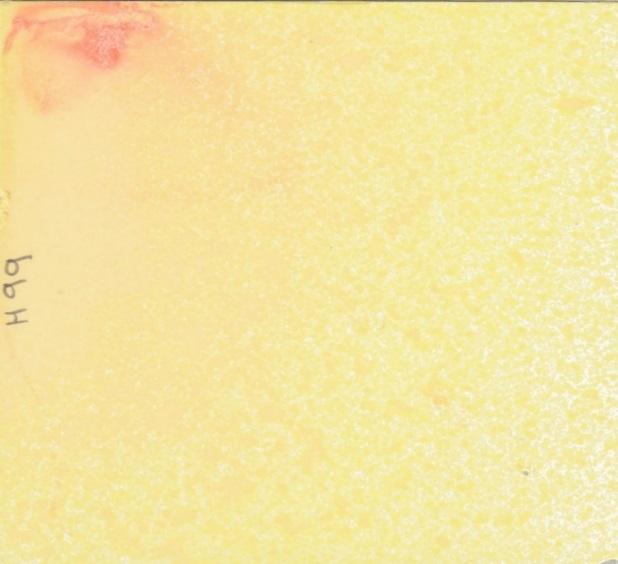

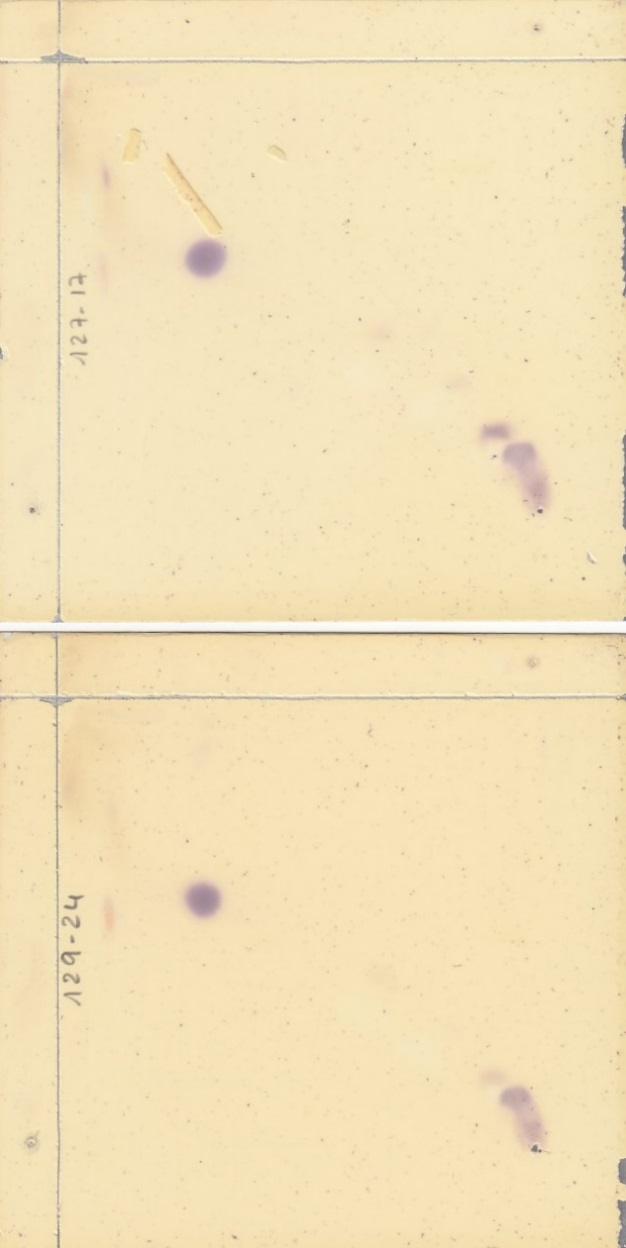

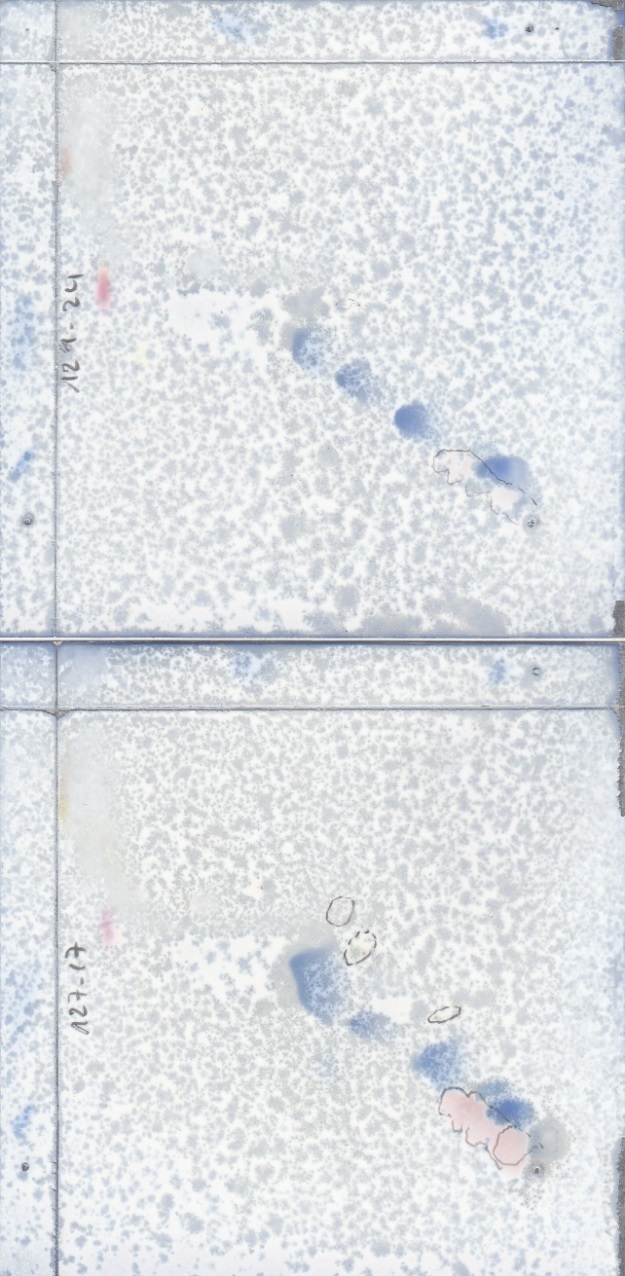


GPL1

GPL2

GL

GPL1

GPL2

PI

PG

DPG

E

D

C

ADI 127-17^T^

ADI 127-17^T^

ADI 127-17^T^

**Figure S4** Two-dimensional TLC plates of polar lipids extracted from *Actinoalloteichus fjordicus* GBA 129-24 stained with (A) molybdatophosphoric acid (Sigma P1518) to identify total lipid content, (B) 0.2 % Ninhydrin reagent to detect amino-groups, (C) Molybdenum blue (Sigma 119KG123) to identify phospho-groups, (D) α-Naphtho-sulphuric acid to detect glyco-groups, (E) Dragendorff reagent to identify choline-groups. Key: DPG, diphosphatidylglycerol; PG, phosphatidylglycerol; PI, phosphatidylinositol; GPL, glycophospholipid; GL, glycolipid and L_1-2_, lipid. Solvent 1: chloroform: methanol: distilled water (65:25:4); Solvent 2: chloroform : glacial acetic acid: methanol: distilled water (80:12:15:4).


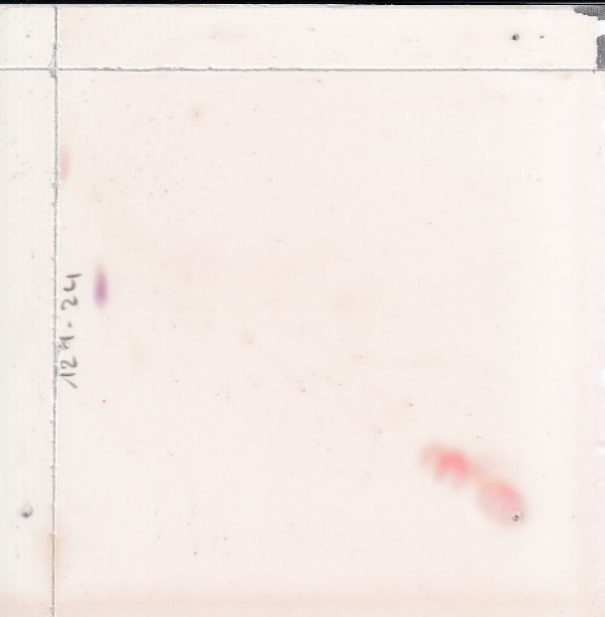


GBA 129-24

A


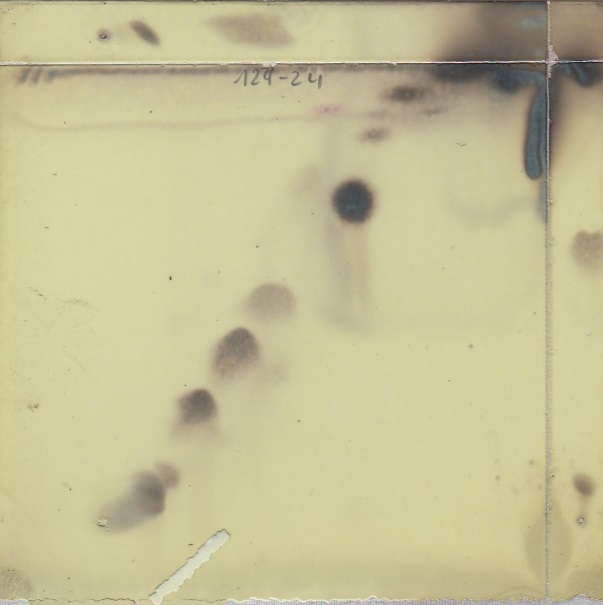


PI

GPL1

GL

PG

DPG

GBA 129-24

GPL2

L1

L2

B

129-24


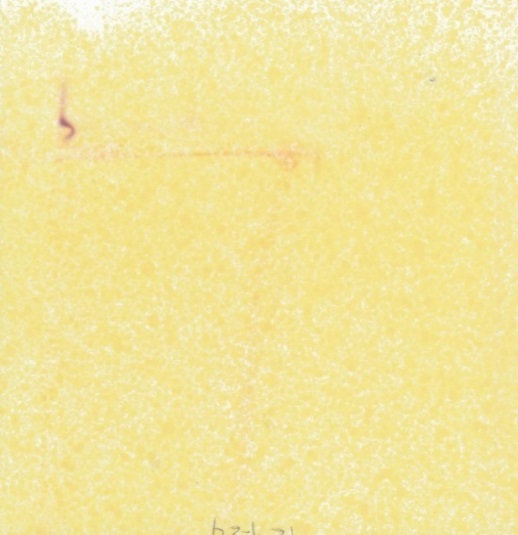

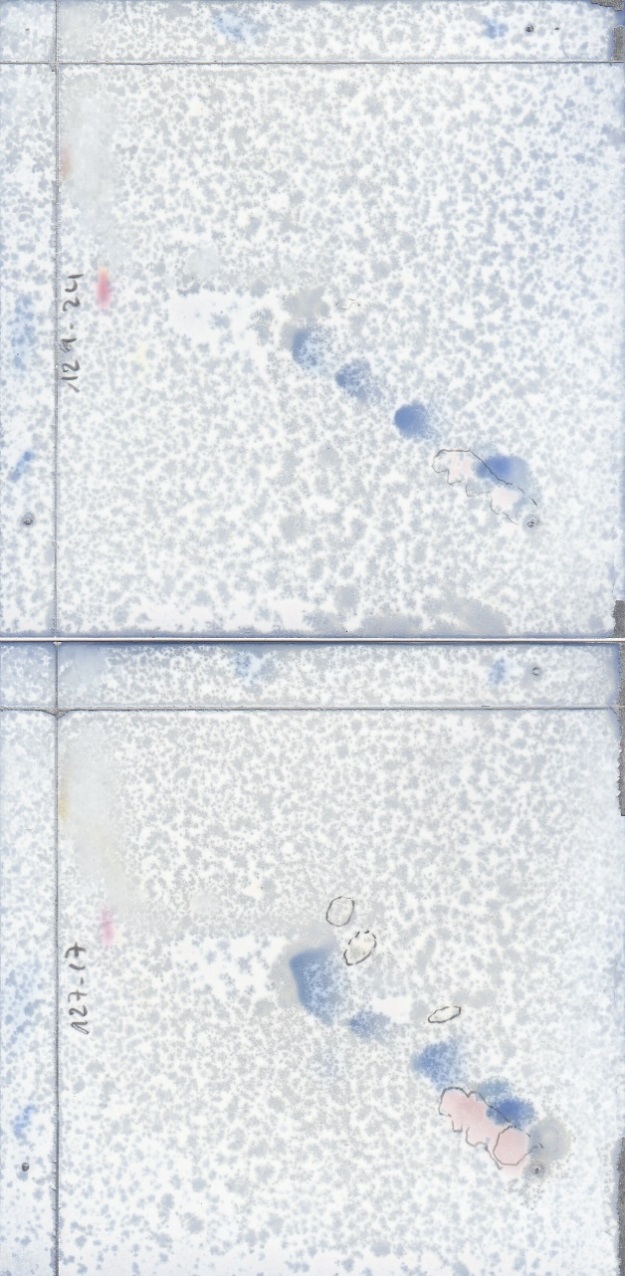

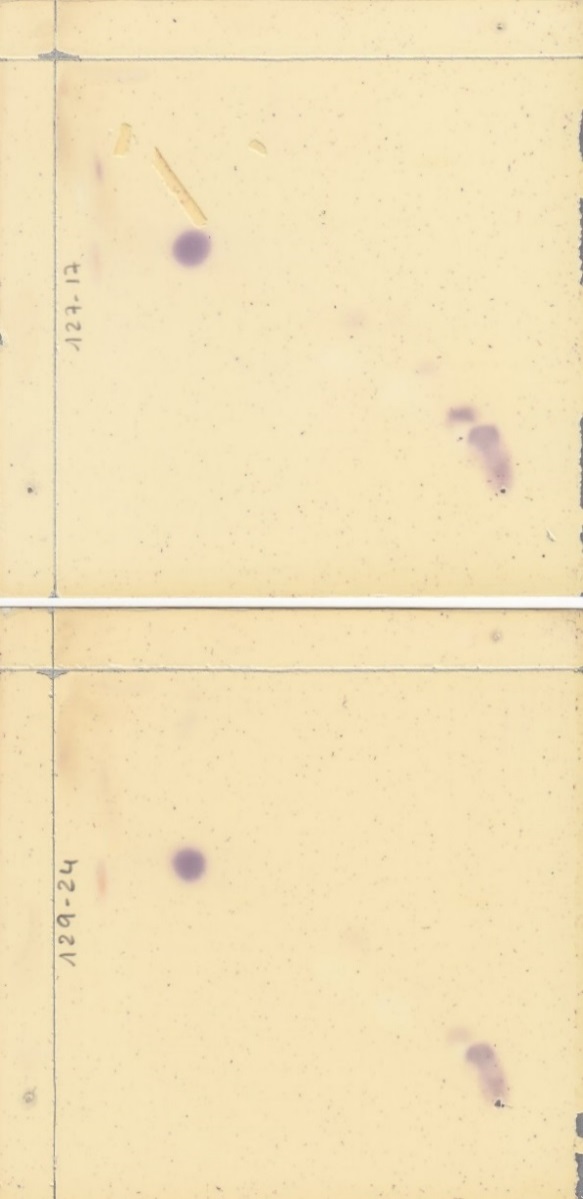


GPL1

GPL2

GL

GPL1

GPL2

PI

PG

DPG

GBA 129-24

GBA 129-24

GBA 129-24

E

D

C
